# Critical appraisal of international guidelines for the screening and treatment of asymptomatic peripheral artery disease: a systematic review

**DOI:** 10.1186/s12872-018-0960-8

**Published:** 2019-01-15

**Authors:** Qinchang Chen, Lingling Li, Qingui Chen, Xixia Lin, Yonghui Li, Kai Huang, Chen Yao

**Affiliations:** 1 Department of Vascular Surgery, the First Affiliated Hospital, Sun Yat-sen University, No.58 Zhongshan Road 2, Guangzhou, China; 2 Medical Intensive Care Unit, the First Affiliated Hospital, Sun Yat-sen University, No.58 Zhongshan Road 2, Guangzhou, China; 3 Department of Vascular Surgery, Sun Yat-sen Memorial Hospital, Sun Yat-sen University, No.33, Yingfeng Road, Guangzhou, China

**Keywords:** Peripheral artery disease, Guidelines, Screening, Treatment

## Abstract

**Background:**

Peripheral artery disease (PAD) is often asymptomatic but increases the risk of developing cardiovascular events. Due to the uncertainties regarding the quality of related guidelines and a lack of clear-cut evidence, we performed a systematic review and critical appraisal of these guidelines to evaluate their consistency of the recommendations in asymptomatic PAD population.

**Methods:**

Guidelines in English between January 1st, 2000 to December 31th, 2017 were screened in databases including Medline via PubMed, EMBASE, the G-I-N International Guideline Library, the National Guidelines Clearinghouse, the Canadian Medication Association Infobase and the National Library for Health. Those guidelines containing recommendations on screening and treatment for asymptomatic PAD were included, and three reviewers evaluated the quality of the guidelines using Appraisal of Guidelines Research and Evaluation (AGREE) II instrument. Related recommendations were then fully extracted and compared by two reviewers.

**Results:**

Fourteen guidelines were included finally and the AGREE scores ranged from 39 to 73%. Most of included guidelines scored low in Rigor of development and Editorial independence, and only two guidelines (ACCF/AHA, AHA/ACC) reached the standard on Conflict of Interest from Institute of Medicine (IOM). Eight guidelines recommended screening at different strength while the others found insufficient evidence or were against screening. Conflicting recommendations on treatment were found in the target value of the lipid lowering and antiplatelet therapy. The treatment policies in three guidelines (BWG, CEVF, ESC) appeared more aggressive, but they had low transparency between guideline developer and industry or did not reach the standard of IOM.

**Conclusions:**

Current guidelines on asymptomatic PAD varied in the methodological quality and fell short of the standard in the rigor of development and editorial independence. Conflicting recommendations were found both on the screening and treatment. More effort is needed to provide clear-cut evidences with high quality and transparency among guideline developer and industry.

**Electronic supplementary material:**

The online version of this article (10.1186/s12872-018-0960-8) contains supplementary material, which is available to authorized users.

## Introduction

Peripheral arterial disease (PAD) is defined as an atherosclerotic process that leads to stenosis and occlusion in non-cerebral and non-coronary arteries [1]. More than 200 million patients worldwide have PAD and the incidence of PAD has increased to nearly 20% in people over 70 [2, 3]. Following coronary heart disease and stroke, PAD has become the third cause of atherosclerotic vascular morbidity [2]. Further prevalence data demonstrates that the number of asymptomatic PAD patients is several times larger than that of the PAD patients with intermittent claudication [1, 4]. Though no obvious clinical symptoms, asymptomatic PAD patients still have a similar risk of premature mortality to that of symptomatic PAD patients, and it is much higher than that of those without PAD [5]. Early detection and treatment of asymptomatic PAD not only prevent its progression, but also lower the risk of developing cardiovascular events, such as myocardial infarction and stroke.

However, sufficient attention has not been paid to asymptomatic PAD, though several studies have been conducted to assess the value of screening for PAD patients [6, 7]. But there was no randomized controlled trials (RCTs) that evaluate the benefits of screening for asymptomatic PAD only, which might lead to different judgement and conflicting recommendations on screening for asymptomatic PAD. The lack of convincing evidence also affects clinical decisions on the treatment for asymptomatic PAD. Thus, the recommendations on screening and treatment for asymptomatic PAD might be impacted by conflicts of interest since transparency among guideline writers was rather low [8, 9]. According to a research in opioid for chronic pain, the organizations funded by opioid manufacturers appeared to oppose to draft guidelines on prescribing opioids, which is worthy of note [10].

In this study, we aimed to systematically appraise the guidelines on the screening and treatment for asymptomatic PAD and find out the agreements and the differences in the recommendations.

## Materials and methods

The systematic review was performed according to the Cochrane methodology [11]. Clinical practice guidelines were defined as statements that contained recommendations with an objective to optimize patient care [12]. There were four steps in the process of the systematic review, including searching for guidelines, selecting guidelines according to specific criteria, appraising the quality of guidelines, and synthesizing recommendations.

### Search strategies

A systematic search was performed to identify relevant guidelines containing the recommendations on management of asymptomatic PAD. We searched the Medline via PubMed and the EMBASE databases. Four guideline-related databases were also searched, including the Guidelines International Network (G-I-N) International Guideline Library, the National Guidelines Clearinghouse (United States), the Canadian Medical Association Infobase (Canada) and the National Library for Health (United Kingdom). The search was limited to guidelines published from January 1st, 2000 to December 31th, 2017. Details on the search strategies was provided in the Additional file 1: Table S1.

### Selection criteria

Guidelines which met following criteria were selected. (1) The target population of the guideline includes asymptomatic PAD patients; (2) the guideline contains recommendations on the screening for asymptomatic PAD and/or treatment for asymptomatic PAD, including but not limited to exercise, pharmacological treatment, surgical treatment; (3) the guideline is available online; (4) the guideline is written in English; (5) the guideline is developed by related national or international academic organizations. Guidelines were excluded out of following reasons: (1) the guideline was not the latest version on the same topic and population; (2) the topic was only mentioned in the guidelines.

### Quality appraisal of the guidelines

Three appraisers (QCC, LLL, CY) assessed the quality of the selected guidelines using the Appraisal of Guidelines Research and Evaluation (AGREE) II instrument. The AGREE II instrument is a 23-item tool with international certification which serves to evaluate the six domains of methodological quality of a guideline, including scope and purpose, stakeholder involvement, rigor of development, clarity of presentation, applicability, and editorial independence [13] (Additional file 2: Table S2). Each item was scored on a scale of 1 (strongly disagree) to 7 (strongly agree) by two appraisers (QCC, LLL). Each appraiser calculated a total score of each domain by adding up scores of all the items in a domain. If scores of the same item differed by more than 1 point between the two appraisers, a consensus meeting would be held to settle the dispute, during which the two appraisers explained the reason for their scores in sequence and then a third appraiser (CY) determined the final score of the item. After that, the obtained score was transformed to a percentage score using the following formula [14]:

$$ \frac{\mathrm{Obtained}\ \mathrm{score}-\mathrm{Minimum}\ \mathrm{possible}\ \mathrm{score}}{\mathrm{Maximum}\ \mathrm{possible}\ \mathrm{score}-\mathrm{Minimum}\ \mathrm{possible}\ \mathrm{score}}\ast 100\% $$

Then each guideline was given a recommendation according to its percentage score. If most (4 or more) domains scored over 60%, a guideline would be regarded as “strongly recommended for use in practice”; if scores of most domains (4 or more) ranged 30–60%, the guideline would be regarded as “recommended for use with some modification”; if most of the domains (4 or more) scored less than 30%, the guideline would be regarded as “not recommended for use in practice”.

### Recommendations synthesis

To examine the consistency of specific recommendations, we used a data extraction form (Additional file 3: Table S3) as previously described [15] to collect some important information from the guidelines, including years of publication, countries/regions, organizations, funding sources, and recommendations on the screening and/or treatments for asymptomatic PAD. The extractions were performed by one reviewer (KH) and validated by another reviewer (QGC). Then the consistency of specific recommendations across different guidelines were assessed and the financial relationships between organizations that produced the guidelines and the biomedical industry were also analyzed.

## Results

### Search results

**Fig. 1:**
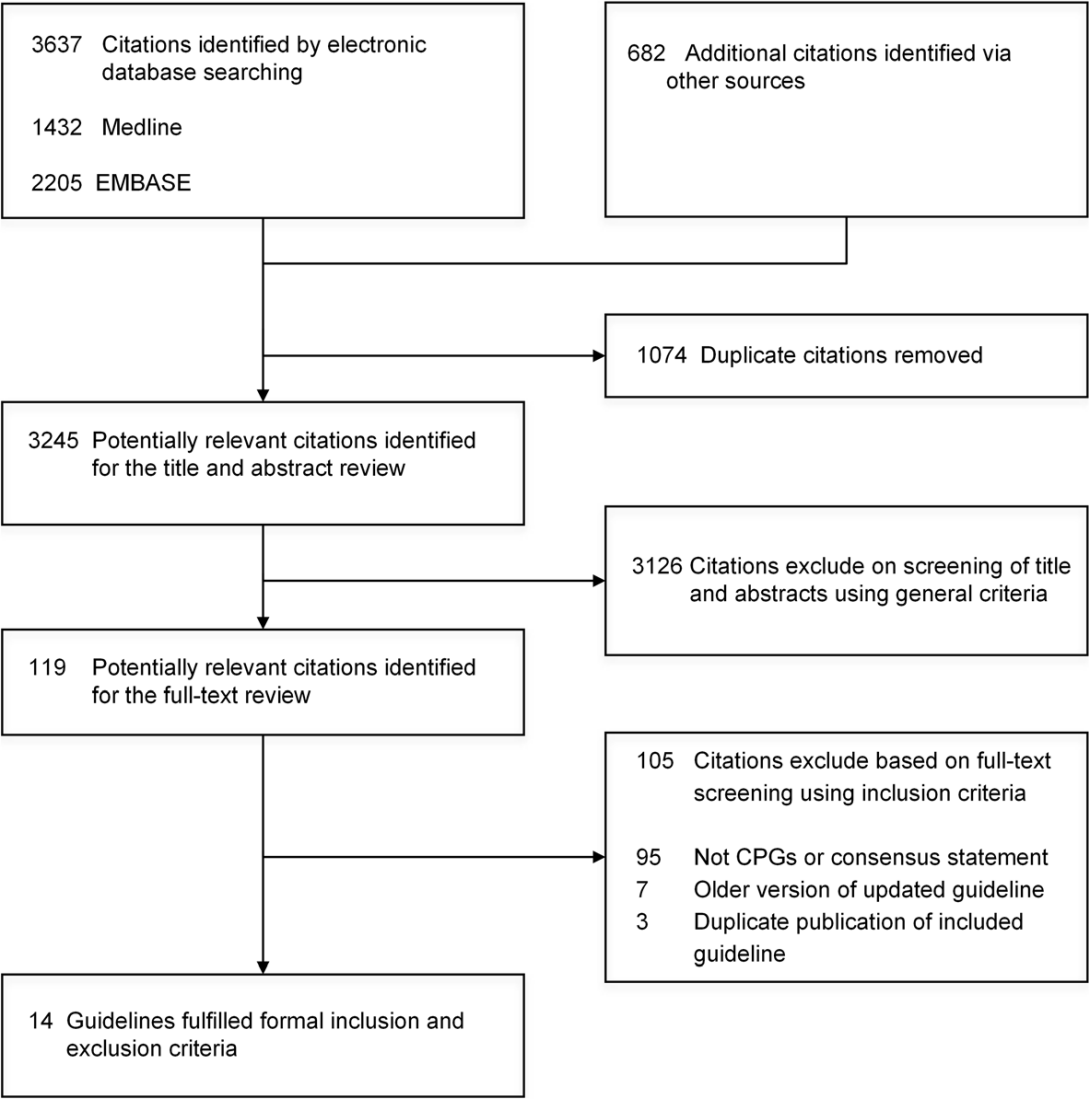
Flow diagram of the identification process for guidelines on screening and treatment in asymptomatic peripheral artery disease

Three thousand two hundred forty-five citations were identified and 3126 citations were excluded after screening the titles and abstracts (Fig. 1). The remaining 119 citations were fully assessed through full texts and 105 citations were excluded because they were not clinical practice guidelines or consensus statements, or they were duplicated publications or older version of updated guidelines. Finally, 14 guidelines pertaining to the management of asymptomatic PAD were included [16–29].

### Characteristics of the guidelines

Characteristics of the included guidelines were presented in Table 1. The guidelines were published between 2007 and 2017. Among them, 6 were developed by USA [16–18, 26, 28, 29], 2 were jointly developed by several European countries [20, 21], and 2 by international organizations named International Working Group on the Diabetic Foot (IWGDF) [24] and Trans-Atlantic Inter-Society Consensus (TASC) [27]. The other four guidelines were formulated in Belgium [19], Germany [22], Italy [23] and Korea [25]. Most guidelines contained recommendations on screening and treatment for asymptomatic PAD but three included recommendations on treatment only [17, 23, 25]. Only one guideline specifically targeted the asymptomatic PAD patients [26]. Seven guidelines reported that systematic literature reviews for the evidence base were performed before developing the guidelines [17, 18, 21, 22, 24–26]. Two guidelines reported that funding came from the pharmaceutical companies [16, 21], one from the government [28], one from both government and commercial companies [17], two received no commercial sponsorship [18, 25]; the others did not report the funding sources. Nine guidelines did not report any information about the conflict of interest (COI) in the chairman or the other members [19, 20, 22–25, 27–29].

**Table 1:** Characteristics of Included Guidelines of Asymptomatic PAD

	Year	Country	Target population	AGREE Rigor score	Evidence base	Funding source	COI of Chairman	COI of members
ACCF AHA	2013	USA	Management of PAD	60	NS	Pharmaceutical company	NO	1/16
ACCP	2012	USA	Antithrombotic therapy	59	Systematic literature review	Government funding; Funding Unrestricted educational grant from pharmaceutical company	NO	6/11
AHA ACC	2016	USA	Management of PAD	70	Systematic literature review	No commercial sponsorship	No	1/21
BWG	2007	Belgium	Diagnosis and treatment of PAD	59	NS	NS	NS	NS
CEVF	2013	Europe	Management of IC	39	Consensus statement	NS	NS	NS
ESC	2017	Europe	Diagnosis and treatment of PAD	70	Systematic literature review	Pharmaceutical company	1/2	17/23
GSA	2016	Germany	Diagnosis and treatment of PAD	61	Systematic literature review	NS	NS	NS
ISD	2014	Italy	Treatment of PAD in diabetes	60	Consensus statement	NS	NS	NS
IWGDF	2016	International	Management of foot ulcer in diabetes	59	Systematic literature review	NS	NS	NS
KSIR	2015	Korea	Interventional recanalization of PAD	72	Systematic literature review	No commercial sponsorship	NS	NS
SVS	2014	USA	Management of asymptomatic PAD	73	Systematic literature review	NS	1/2	7/11
TASC	2007	International	Management of PAD	70	Consensus statement	NS	NS	NS
USPSTF	2013	USA	Screening of PAD	62	NS	Government funding	NS	NS
WHS	2014	USA	Treatment of arterial ulcer	62	NS	NS	NS	NS

*Abbreviations*: *ACCF AHA* American College of Cardiology Foundation, American Heart Association Task Force, *AGREE* Appraisal of Guidelines Research and Evaluation, *AHA ACC* American College of Cardiology, American Heart Association Task Force, *BWG* Belgian Working Group, *CEVF* Central European Vascular Forum, *COI* Conflict of Interest, *ESC* European Society of Cardiology, *GSA* German Society of Angiology, *IC* Intermittent Claudication, *ISD* Italian Societies of Diabetes, *IWGDF* International Working Group on the Diabetic Foot, *KSIR* Korean Society of Interventional Radiology, *NS* Not Stated, *PAD* Peripheral Arterial Disease, *SVS* Society for Vascular Surgery, *TASC* Trans-Atlantic Inter-Society Consensus, *USPSTF* US Preventive Services Task Force, *WHS* Wound Healing Society

### Guideline appraisal

**Fig. 2:**
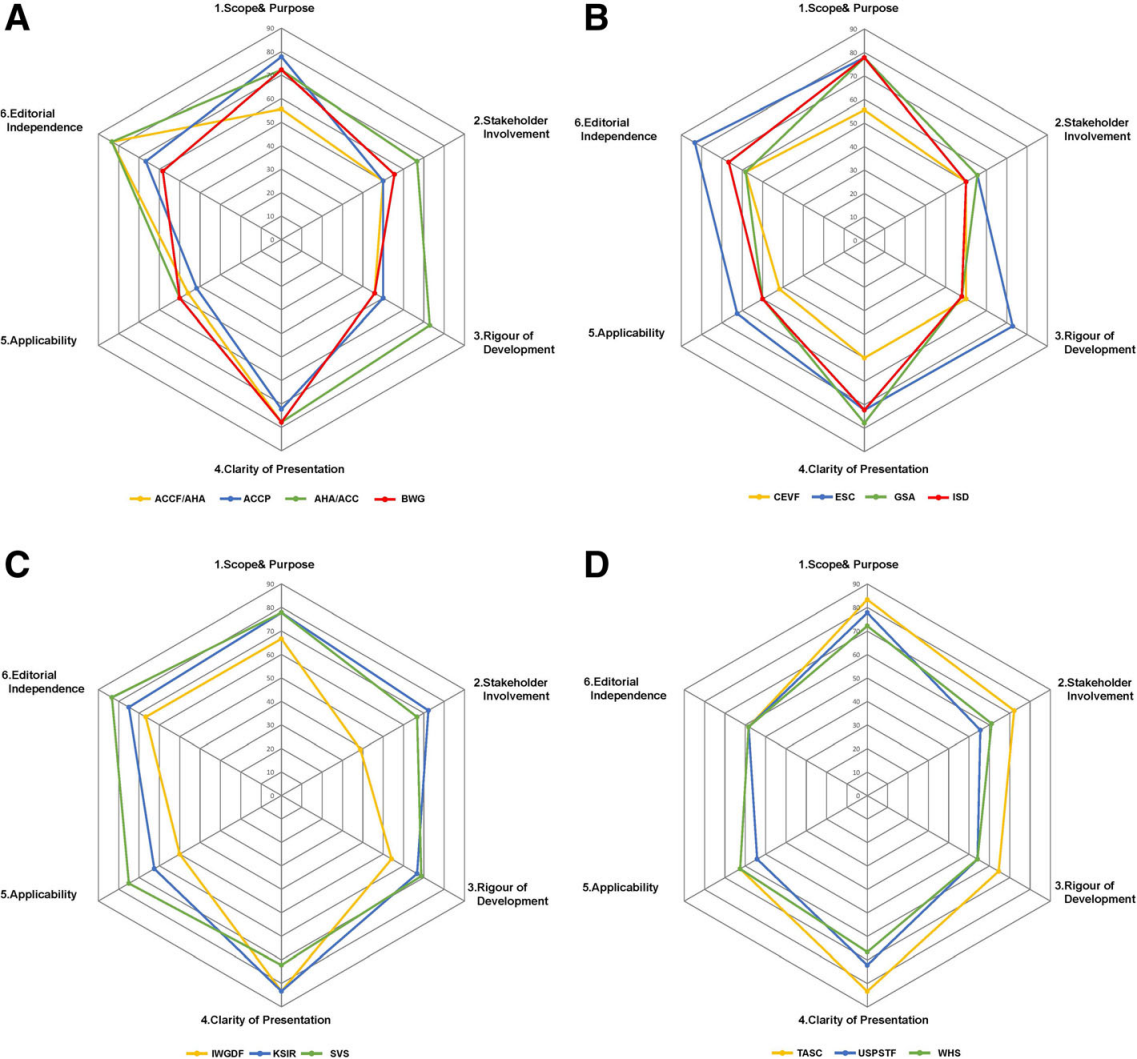
Final Domain Scores. AGREE II scores are plotted for each guideline for comparison. The higher percentage meant the better quality in the domain and was mapped towards the outer perimeter (closer to 100%). Abbreviations: ACCF AHA, American College of Cardiology Foundation, American Heart Association Task Force; AGREE, Appraisal of Guidelines Research and Evaluation; AHA ACC, American College of Cardiology, American Heart Association Task Force; BWG, Belgian Working Group; CEVF, Central European Vascular Forum; COI, Conflict of Interest; ESC, European Society of Cardiology; GSA, German Society of Angiology; IC, Intermittent Claudication; ISD, Italian Societies of Diabetes; IWGDF, International Working Group on the Diabetic Foot; KSIR, Korean Society of Interventional Radiology; NS, Not Stated; PAD, Peripheral Arterial Disease; SVS, Society for Vascular Surgery; TASC, Trans-Atlantic Inter-Society Consensus; USPSTF, US Preventive Services Task Force; WHS, Wound Healing Society

The final scores of six domains in each guideline were shown in Fig. 2. In order to visually gauge the strength and weakness of each domain between guidelines, we selected radar chart rather than histogram to present the result of guideline appraisal. The higher percentage meant the better quality in the domain and was mapped towards the outer perimeter (closer to 100%). As it is shown in the graph, the guidelines from AHA/ACC, ESC, KSIR, SVS and TASC had relatively higher scores in most domains [17, 21, 25–36], [37], and most guidelines had higher scores in the domain 4 (Clarity of presentation). However, several guidelines scored low in domain 3 (Rigor of development) and domain 6 (Editorial independence). Some guidelines did not use systematic review protocol and there was still too low transparency between guideline writers and industry.

Six guidelines were regarded as “strongly recommended for use in practice”, namely AHA/ACC, ESC, GSA, KSIR, SVS, TASC and WHS [18, 21, 22, 25–27]. The remaining guidelines were regarded as “recommended for use with some modification” and no guideline is regarded as “not recommended for use in practice”.

### Recommendations on approaches to screening

Ten guidelines contained the recommendations on screening for asymptomatic PAD [16, 18–22, 24, 28, 29]. The key recommendations were shown in Table 2. Guidelines differed slightly in the strength of recommendations on screening for asymptomatic population. Five guidelines [19, 21, 22, 24, 29] strongly recommended the screening while three [16, 20, 26] supported screening at the strength from moderated to strong. However, USPSTF guideline [28] did not provide the decision on screening because of insufficient evidence and AHA/ACC guideline [18] considered that screening harmed the asymptomatic population. Apart from AHA/ACC guideline [18] considering non-invasive angiography, all the remaining guidelines recommended the invasive test, especially ABI test. Five guidelines [16, 19, 20, 22, 24] provided the normal range of ABI and made an agreement (0.9–1.3). The difference of target population was the age of the patients without elevated cardiovascular risk. Targeted ages of three guidelines [19, 20, 26] were more than 70 years old while that of ESC guideline was 65 years old [21]. In terms of the further test, only four guidelines [16, 20, 22, 24] gave recommendations while the others did not. Among them, three guidelines recommended exercise ABI while GSA guideline considered no further test.

**Table 2:** Summary of recommendations on screening of asymptomatic PAD

	Contents	Strength of recommend-ations	Screening tests	Normal/abnormal ABI	Target population	Further testing
ACCF AHA	Management of PAD	For (moderate-strong)	ABI	normal ABI (0.91 to 1.30)		Exercise ABI if ABI is normal; TBI or PVR if ABI >1.30
AHA ACC	Management of LEAD	Harm	Invasive and noninvasive angiography (ie, CTA, MRA)			
BWG	Diagnosis and treatment of PAD	For (strong)	Clinical examination including ABI	ABI < 0.9 suggests abnormal	•Subjects from 50 to 69 years of age with diabetes, smoking, hypertension, dyslipidemia; •Subjects older than 70 years of age; • Subjects with history of other CV disease	
CEVF	Management of IC	For (moderate-strong)	Standard clinical review including ABI	normal ABI (0.91 to 1.30) ABI < 0.9 suggests abnormal	• Adults older than 50 years with atherosclerosis risk factors •Adults greater than 70 years.	Exercise ABI if at risk for PAD; ABI is normal; without claudication symptoms; no other AS evidence
ESC	Diagnosis and treatment of PAD	For (strong)	ABI		•Men and women aged >65 years •Men and women aged <65 years classified at high CV risk •Men and women aged >50 years with family history for LEAD	
GSA	Diagnosis and treatment of PAOD	For (strong)	Clinical examination of the feet, including basic evaluation, history and PE, resting ABI	All pulses palpable+ ABI 0.9 -1.3 suggested asymptomatic PAOD.		No further test
IWGDF	Diagnosis, prognosis and management of PAD in patients with foot ulcers in diabetes	For (strong)	Bedside non-invasive tests	ABI <0.9 considered abnormal		Largely exclude PAD are ABI 0.9–1.3, TBI ≥0.75 and with TPDAW
SVS	Management of asymptomatic atherosclerotic occlusive disease of the lower extremities and claudication	For (moderate)	ABI		For asymptomatic individuals who are at elevated risk, such as those aged >70, smokers, diabetic patients, those with an abnormal pulse examination, or other CV disease	
USPSTF	Screening for PAD	Insufficient evidence	ABI			
WHS	Management of arterial ulcers	For (strong)	Audio handheld Doppler waveforms; Triphasic pulse.			

*Abbreviations*: *ABI* Ankle Brachial Index, *ACCF AHA* American College of Cardiology Foundation, American Heart Association Task Force, *AGREE* Appraisal of Guidelines Research and Evaluation, *AHA ACC* American College of Cardiology, American Heart Association Task Force, *AS* atherosclerosis, *BMI* Body Mass Index, *CEVF* Central European Vascular Forum, *CTA* Computed Tomography Angiography, *CV* Cardiovascular, *ESC* European Society of Cardiology, *GSA* German Society of Angiology, *IC* Intermittent Claudication, *IWGDF* International Working Group on the Diabetic Foot, *LEAD* Lower Extremity Arterial Disease, *MRA* Magnetic Resonance Angiography, *PAD* Peripheral Arterial Disease, *PAOD* Peripheral Arterial Occlusive Disease, *PE* Physical Examination, *SVS* Society for Vascular Surgery, *TASC* Trans-Atlantic Inter-Society Consensus, *TBI* Toe Brachial Index, *TPDAW* Triphasic Pedal Doppler Arterial Waveforms, *USPSTF* US Preventive Services Task Force, *WHS* Wound Healing Society

### Recommendations on approaches to treatment

Table 3 showed the recommendations from eleven guidelines for the medical management of asymptomatic PAD, including the secondly prevention and surgical treatment [16–23, 25–27]. The secondly prevention included smoking cessation, healthy diet, lipid lowering, hypertension treatment, diabetes treatment and antiplatelet therapy. Smoking cessation was recommended by five guidelines and there was no different recommendation [16, 19–21, 26]. For lipid lowering, two guidelines [16, 20] recommended to lower lipid but did not provide a target value. Three guidelines [19, 21, 27] recommended using stains to lower lipid but with different target value. ESC guideline [21] suggested LDL-C less than 70 mg/L while the others [19, 27] recommended 100 mg/L. Hypertension treatment was recommended in four guidelines [16, 19–21]. ACCF/AHA guideline [16] regarded ACEI as the ideal drug and BWG guideline [19] provided the target value. Diabetes treatment was recommended in four guidelines [16, 19–21] and BWG guideline [19] provided more detail information. Antiplatelet therapy was recommended in seven guidelines [16–21, 23]. The controversy was at the administration dosage. ACCP and ISD guidelines [17, 23] considered 75-100 mg for treatment while BWG guideline [19] recommended 75-150 mg. Arterial reconstruction was not recommended in four guidelines [16, 22, 25, 26] and there was not controversial recommendation.

**Table 3:** Summary of recommendations on treatments of asymptomatic PAD

	Objective	Smoking cessation	Healthy diet and physical activity	Lipid lowering	Hypertension treatment	Diabetes treatments	Antiplatelet therapy	Arterial reconstruction
ACCF AHA	Management of PAD	Recommended		Recommended	Recommended; ACEi may be considered	Recommended	Asymptomatic LEAD: Indicated; Asymptomatic PAD with ABI (0.91-0.99): Usefulness is not well established	Not indicated as prophylactic therapy
ACCP	Antithrombotic Therapy in PAD						Asymptomatic PAD: Aspirin 75 to 100 mg daily	
AHA ACC	Management of LEAD						Asymptomatic PAD (ABI≤0.90): Reasonable; Asymptomatic PAD with ABI (0.91-0.99): Uncertain	
BWG	Diagnosis and treatment of PAD	Recommended		Stains: LDL-C< 100 mgdl If high risk, LDL-C < 70 mgdl	BP<140/90 mmHg; BP <130/80 mmHg if diabetes renal insufficiency	HbA1c < 7%, as low as possible	All PAD patients: ASA (75-150 mg) ASA is contra-indicated new CV event under ASA treatment: Clopidogrel (Plavix® 75 mg)	
CEVF	Management of IC	Recommended		Recommended	Recommended	Recommended	Asymptomatic LEAD: Indicated	
ESC	Diagnosis and treatment of PAD	Recommended	Recommended	Stains LDL-C <70mgdL; If initial LDL-C level is 70-135mgdL, decreased by >50%	Recommended	Recommended	Not routinely indicated in isolated asymptomatic LEAD	
GSA	Diagnosis and treatment of PAOD							Not indicated for Asymptom-atic PAOD
ISD	Treatment of PAD in diabetes						Diabetic + aged >50 years+ asymptomatic PAD: Long-term daily aspirin (75-100 mg)	
KSIR	Interventional Recanalization of Lower Extremity Arteries							Not require prophylactic Recanalization
SVS	Management of asymptomatic atherosclerotic occlusive disease of the lower extremities and claudication	Recommended (repeatedly until tobacco use has stopped)						Against invasive treatments for PAD in the absence of symptoms
TASC	Management of PAD			LDL-C <100mgdl				

*Abbreviations*: *ABI* Ankle Brachial Index, *ACEI* Angiotensin-converting Enzyme Inhibition, *ACCF AHA* American College of Cardiology Foundation, American Heart Association Task Force, *ACCP* American College of Chest Physicians, *AGREE* Appraisal of Guidelines Research and Evaluation, *AHA ACC* American College of Cardiology, American Heart Association Task Force, *ASA* Acetylsalicylic Acid, *BP* Blood Pressure, *BWG* Belgian Working Group, *CEVF* Central European Vascular Forum, *CV* Cardiovascular, *ESC* European Society of Cardiology, *GSA* German Society of Angiology, *HbA1c* Glycosylated Hemoglobin, *IC* Intermittent Claudication, *ISD* Italian Societies of Diabetes, *LDL-C* Low-density Lipoprotein Cholesterol, *LEAD* Lower Extremity Arterial Disease, *KSIR* Korean Society of Interventional Radiology, *PAD* Peripheral Arterial Disease, *PAOD* Peripheral Arterial Occlusive Disease, *SVS* Society for Vascular Surgery, *TASC* Inter-Society Consensus for the Management of Peripheral Arterial Disease

## Discussion

As far as we know, this is the first guideline appraisal on asymptomatic PAD. In summary, 14 guidelines were identified which covered the management of asymptomatic PAD. Seven guidelines lacked a systematic literature review and nine reported too little information about COI, resulting in low AGREE scores for rigor of development and editorial independence. Ten guidelines contained recommendations about screening with five guidelines at strong strength, three at moderate to strong strength, while others being against it or finding insufficient evidence. ABI test was generally recommended, sometimes with other non-invasive examinations. Its target group is the middle-aged population with increasing cardiovascular risk and elderly. Smoking cessation, hypertension treatment and diabetes therapy were also generally recommended while arterial reconstruction was not indicated. Lipid lowering and antiplatelet therapy were recommended by some guidelines but with controversy in target value.

PAD is a condition with significant morbidity `and mortality, affecting nearly 200 million people worldwide [1]. Notably, nearly 90% of these individuals are asymptomatic [30]. However, insufficient attention has been paid to these populations. Among the 14 included guidelines, only SVS guideline [26] specially focused on the asymptomatic individuals. Conflicting recommendations were observed both on screening and treatment. For screening for asymptomatic patients, several guidelines supported it at different strength while two were against the screening or considered evidence being insufficient to recommend. AHA/ACC guideline [18] used invasive and non-invasive angiography as screening methods and is the only included guideline which put screening in the “harm” category. It is worth noting that AHA/ACC guidelines met the threshold for an AGREE II score of “recommended for use in clinical practice”, making its recommendations appear to be reliable. The recommendation of screening was mainly on the basis of two studies [31, 32]. Catalano M et al. [31] conducted a randomized, placebo-controlled, double-blind clinical trial to assess the medical efficacy of aspirin and a high dose antioxidant vitamin combination in PAD patients to reduce the risk of vascular event. However, not all the patients were asymptomatic PAD and 76% of them were with type 2 diabetes. Minar et al. [32] performed a randomized trial to investigate the effect of different dosage of aspirin in PAD population, rather than the asymptomatic patients. Till now, there was no randomized controlled trial (RCT) directly analyze the effect of screening for PAD in terms of some important outcomes, such as mortality, which is most important for guideline developers to provide recommendations. Since no RCT was avaiable, meta-analyses have been the main source of evidences, but it is not able to reclassify the asymptomatic participants from intermediate to high cardiovascular risk. Fares et al. [30] conducted a meta-analysis in 19 studies and observed large inconsistency in results, which demonstrated the heterogeneity in the risk of the populations and a range of cardiovascular risk among asymptomatic patients. The absence of evidence of high quality and the lager heterogeneity in the selected population might account for the conflicting recommendations in screening.

In terms of treatment, the conflicting recommendations were mainly observed in the target value of lipid lowering and antiplatelet therapy. As it is shown in Table 3, the recommendations from BWG, CEVF and ESC appeared a more aggressive treatment policy, which might be explained by the financial relationship between developers and pharmaceutical industry. In 2011, Institute of Medicine published the standard on conflict of interest, requiring that there should be no COI on committee chairs and less than 50% of committee members having commercial relationship [33]. According to the standard, only two guidelines (ACCF/AHA, AHA/ACC) met the requirement, indicating that the transparency among guideline writers and industry became a problem. When there is no clear-cut evidence at high quality, the decision may be easily affected by the commercial relationship. The aggressive policy to lower the target value increased the dosage of stain or antiplatelet drug, which might be related to the interest of the industry. Concerning the incomplete information in the included guidelines about potential COI, a transparent development process should be highlighted to ensure that the clinical guidelines establishing appropriate care for asymptomatic PAD patients. Apart from the pharmaceutical treatment and arterial reconstruction, smoking cessation was recommended by five guidelines [16, 19–21, 26]. Smoking has been demonstrated as an important risk factor of PAD [1]. The recommendation of smoking cessation was at level of evidence B in three guidelines [16, 20, 21] and at level of evidence A [26] in one guideline. Healthy diet and physical activity were only recommended by ESC guideline [21] while they were not mentioned by others. The recommendation was at the level of evidence C but supporting evidence was not provided. In a word, RCTs in asymptomatic PAD patients should be performed to directly investigate the effect of lifestyle modification.

To make up for the lack of clear-cut evidence as described above and decrease the impact of financial relationship with pharmaceutical industry, RCTs of PAD screening versus no screening should be performed in asymptomatic PAD patients. After detection of PAD, interventions are advocated by some included guidelines, which mainly recommend lifestyle intervention, lipid lowing and antiplatelet therapy. However, whether these interventions would be effective in the asymptomatic PAD population is still difficult to answer. The dosage of antiplatelet therapy and target value are also controversy in different guidelines. Without the clear-cut evidence for treatment, the recommendation for medication is more sensitive to conflicts of interest with pharmaceutical industry and biased toward a more aggressive treatment policy. Thus, RCTs for treatment should also be performed to investigate intervention versus no intervention in asymptomatic population. Once the beneficial effect is confirmed, further prospective studies should be carried out to investigate the suitable dosage or target value.

There were several limitations in our study. Firstly, all the selected guidelines were only in English, resulting in the potential selecting bias. Fortunately, even though their official language was not English, some organizations appeared to publish the guidelines in English version. Secondly, we selected AGREE II as the assessment tool, rather than other appraisal tools, such as the four-item Global Rating Scale (GRS) [34]. Although AGREE II instrument has been recognized and widely used in the guideline appraisal [14, 35, 36], whether results using other appraisal tools are consistent remains unkown. Thirdly, the appraisal using the AGREE instrument was only based on the whole guidelines, rather than specific or individual recommendations. This might weaken the aim of the guideline appraisal which is to provide a whole picture for assessing the quality of reporting recommendations and suggestion on how to improve in the future. Finally, we did not restricted the regions of the included guidelines. Hence, the results may be not that generalizable to readers from the specific region, such as countries with greater or lesser burden of diseases.

## Conclusions

Current guidelines about asymptomatic PAD varied in the methodological quality and fell short of the standard in the rigor of development and editorial independence. The conflicting recommendations were both on the screening and treatment. Increasing effort is needed to provide the clear-cut evidence with high quality and transparency among guideline developers and industry.

## Additional files

Additional file 1: **Table S1.** Search strategies. (DOCX 17 kb)

Additional file 2: **Table S2.** Structure and content of the AGREE instrument. (DOCX 15 kb)

Additional file 3: **Table S3.** Data extraction template. (DOCX 14 kb)

## Abbreviations

ACCF AHA: American College of Cardiology Foundation, American Heart Association Task Force
AGREE: Appraisal of Guidelines Research and Evaluation
AHA ACC: American College of Cardiology, American Heart Association Task Force
BWG: Belgian Working Group
CEVF: Central European Vascular Forum
COI: Conflict of Interest
ESC: European Society of Cardiology
GSA: German Society of Angiology
IC: Intermittent Claudication
ISD: Italian Societies of Diabetes
IWGDF: International Working Group on the Diabetic Foot
KSIR: Korean Society of Interventional Radiology
NS: Not Stated
PAD: Peripheral Arterial Disease
SVS: Society for Vascular Surgery
TASC: Trans-Atlantic Inter-Society Consensus
USPSTF: US Preventive Services Task Force
WHS: Wound Healing Society
