# Supplementary material for: Critical appraisal of international guidelines for the screening and treatment of asymptomatic peripheral artery disease: a systematic review
Source: BMC Cardiovasc Disord. 2019 Jan 15;19:17. doi: 10.1186/s12872-018-0960-8 (PMC6332557; doi:10.1186/s12872-018-0960-8)
Supplement: Supplementary file 1 — Table S1. Search strategies. (DOCX 17 kb) [file 12872_2018_960_MOESM1_ESM.docx]

**Additional file 1: Table S1. Search Strategies**

Search strategies Medline

1. "guideline"[Publication Type]
2. "guidelines as topic"[MeSH Terms]
3. "guideline"[All Fields]
4. 1 OR 2 OR 3
5. " peripheral artery diseases "[Mesh]
6. Arterial Disease, Peripheral
7. Arterial Diseases, Peripheral
8. Disease, Peripheral Arterial
9. Diseases, Peripheral Arterial
10. Peripheral Arterial Diseases
11. Peripheral Artery Disease
12. Artery Disease, Peripheral
13. Artery Diseases, Peripheral
14. Disease, Peripheral Artery
15. Diseases, Peripheral Artery
16. Peripheral Artery Diseases
17. 5 OR 6 OR7 OR 8 OR 9 OR 10 OR 11 OR 12 OR 13 OR 14 OR 15 OR 16
18. “Asymptomatic Diseases” [Mesh]
19. Asymptomatic Disease
20. Disease, Asymptomatic
21. Diseases, Asymptomatic
22. Asymptomatic States
23. Asymptomatic State
24. Presymptomatic Diseases
25. Disease, Presymptomatic
26. Diseases, Presymptomatic
27. Presymptomatic Disease
28. Asymptomatic Conditions
29. Asymptomatic Condition
30. Condition, Asymptomatic
31. Conditions, Asymptomatic
32. Pre-Symptomatic Diseases
33. Disease, Pre-Symptomatic
34. Diseases, Pre-Symptomatic
35. Pre-Symptomatic Disease
36. 18 OR 19 OR 20 OR 21 OR 22 OR 23 OR 24 OR 25 OR 26 OR 27 OR 28 OR 29 OR 30 OR 31 OR 32 OR 33 OR 32 OR 35
37. 4 AND 17 AND 36

Search strategies EMBASE

1. heart disease$.tw.
2. (stroke$ or cerebrovasc$ or cva$).tw.
3. (aort$ adj5 aneurysm).tw.
4. (abdominal adj5 aneurysm).tw.
5. cardiovascular diseases/
6. exp coronary disease/
7. exp cerebrovascular disorders/
8. exp aortic aneurysm/
9. peripheral vascular diseases/
10. heart failure/
11. exp arteriosclerosis/
12. (cardiovascular adj3 disease$).tw.
13. (coronary adj3 disease$).tw.
14. (thoracoabdominal adj5 aneurysm).tw.
15. (arteri$ adj3 (occlusi$ or stenosis)).tw.
16. (peripher$ adj5 (occlusi$ or arteri$ or vascular)).tw. exp practice guideline/
17. Or/1-16
18. Asymptomatic
19. guideline$.tw.
20. consensus.tw.
21. position statement$.tw.
22. exp health care policy/ or exp policy/
23. recommendation$.tw.
24. or/19-23
25. 17 and 18 and 24
