# Supplementary material for: Critical appraisal of international guidelines for the screening and treatment of asymptomatic peripheral artery disease: a systematic review
Source: BMC Cardiovasc Disord. 2019 Jan 15;19:17. doi: 10.1186/s12872-018-0960-8 (PMC6332557; doi:10.1186/s12872-018-0960-8)
Supplement: Supplementary file 2 — Table S2. Structure and content of the AGREE instrument. (DOCX 15 kb) [file 12872_2018_960_MOESM2_ESM.docx]

**Additional file 2: Table S2. Structure and Content of the AGREE II instrument**

The following is adapted from the AGREE II instrument (Brouwers M C, Kho M E, Browman G P, et al. AGREE II: advancing guideline development, reporting and evaluation in health care[J]. Canadian Medical Association Journal, 2010, 182(18): E839-E842.)

| **Domains** | **Content** | **No. of items** |
| --- | --- | --- |
| Scope and purpose | Addresses the overall aim of the guideline, the specific clinical questions and targets patient population | 3 |
| Stakeholder involvement | Addresses the extent to which the guideline represents the views of its intended users (relevant professional groups, patients, target users defined, piloting among target users) | 3 |
| Rigor of development | Addresses the process used to collect and synthesize the evidence, the methods to formulate the recommendations, process for updating the guidelines, external review | 8 |
| Clarity and presentation | Addresses the language and format of the guideline (recommendations are specific and unambiguous, different options for management are presented, key recommendations are identifiable, tools for application are available) | 3 |
| Applicability | Addresses the likely organisational, behavioral, and cost implications of applying the guideline, key criteria for monitoring and/or audit purposes | 4 |
| Editorial independence | Addresses the independence of the recommendations and acknowledgement of possible conflict of interest from the guideline development group | 2 |
