# Supplementary material for: Critical appraisal of international guidelines for the screening and treatment of asymptomatic peripheral artery disease: a systematic review
Source: BMC Cardiovasc Disord. 2019 Jan 15;19:17. doi: 10.1186/s12872-018-0960-8 (PMC6332557; doi:10.1186/s12872-018-0960-8)
Supplement: Supplementary file 3 — Table S3. Data extraction template. (DOCX 14 kb) [file 12872_2018_960_MOESM3_ESM.docx]

**Additional file3: Table S3. Data Extraction Template**

| **Item** | **Entry** |
| --- | --- |
| Guideline Id |  |
| Organization/society |  |
| Name of guideline or consensus statement |  |
| Year of publication |  |
| Funding source |  |
| Country |  |
| Target population |  |
| Definition of VTE Patients with cancer |  |
| Target Users |  |
| Guideline writers |  |
| Guideline Review |  |
| Guideline Update |  |
| Methods Support |  |
| Evidence Base |  |
| Level of Evidence |  |
| Grade recommendations |  |
| Recommendations 1 |  |
| Recommendations 2 |  |
| Recommendations 3 |  |
| Recommendations 4 |  |
| Recommendations 5 |  |
| Recommendations 6 |  |
| Recommendations 7 |  |
| Recommendations 8 |  |
| Recommendations 9 |  |
| Recommendations 10 |  |
